# Protein phosphatases in systemic autoimmunity

**DOI:** 10.1097/IN9.0000000000000056

**Published:** 2025-02-10

**Authors:** Wenliang Pan, Maria G. Tsokos, Wei Li, George C. Tsokos

**Affiliations:** 1 Department of Medicine, Beth Israel Deaconess Medical Center and Harvard Medical School, Boston, MA, USA

**Keywords:** phosphatase, autoimmunity, PP2A, TC-PTP, SHP1, SHP2, PTPN22, DUSP22, CD45, DUSP2

## Abstract

Protein phosphatases play a critical role in maintaining immune homeostasis by regulating various signaling pathways involved in immune cell activation, differentiation, and function. In the context of systemic autoimmune diseases, dysregulation of phosphatase activity contributes to aberrant immune responses, leading to chronic inflammation and tissue damage. This review explores the role of key phosphatases from the protein serine/threonine phosphatase and protein tyrosine phosphatase families that are implicated in systemic autoimmunity. We discuss their diverse roles in immune cell subsets, the mechanisms by which their dysregulation drives autoimmune pathogenesis, and the therapeutic potential of targeting these enzymes.

## 1. Introduction

The human protein phosphatome is composed of 189 known and predicted genes ^[1]^ that encode protein phosphatases classified into three major superfamilies based on their catalytic mechanisms: the protein serine/threonine phosphatases (PSP), protein tyrosine phosphatases (PTP), and haloacid dehalogenase (HAD) superfamilies ^[2–5]^. Each superfamily comprises enzymes that target specific phosphorylated residues, such as phospho-serine/threonine (phospho-Ser/Thr) or phospho-tyrosine (phospho-Tyr), although some enzymes within these groups can act on substrates typically recognized by another group. For example, some phosphatases in the PTP or PSP families may dephosphorylate both phospho-Ser/Thr and phospho-Tyr residues, and some HAD phosphatases may also target phospho-Ser/Thr or phospho-Tyr.

Phosphatases play a crucial role in regulating immune responses by modulating the phosphorylation states of proteins. Dysregulation of phosphatase activity can lead to abnormal immune signaling, contributing to the development of autoimmune diseases. Currently, several phosphatases have been identified as contributors to the pathogenesis of autoimmune diseases, including members of the PSP family, such as protein phosphatase 2A (PP2A), and of the PTP family, such as T-cell PTP (TC-PTP), Src homology-2 domain-containing PTP1 (SHP1), Src homology-2 domain-containing PTP2 (SHP2), PTP non-receptor type 22 (PTPN22), dual specificity phosphatase 22 (DUSP22), dual specificity phosphatase 2 (DUSP2), and CD45 (Table 1). This review highlights the critical roles of these phosphatases in the immune regulation and the development of autoimmune diseases. Further studies into their mechanisms may lead to targeted therapies for autoimmune disorders.

**Table 1:** Protein phosphatases involved in autoimmune diseases.

Phosphatase name	Gene	Class	Clinical evidences in autoimmune diseases
PP2A	*PPP2CA/PPP2CB* *(catalytic*)	PSP	SLE ^[6–13]^
TC-PTP	*PTPN2*	PTP	CD ^[14,15]^, UC ^[15,16]^, T1D ^[17,18]^, celiac disease ^[17,18]^
SHP1	*PTPN6*	PTP	MS ^[19,20]^, RA ^[21]^, emphysema ^[22]^
SHP2	*PTPN11*	PTP	RA ^[23]^, UC ^[14,24]^, CD ^[14,24]^, SLE ^[25,26]^, IBD ^[27]^
PTPN22	*PTPN22*	PTP	T1D ^[28]^, SLE ^[29,30]^, RA ^[29,31,32]^, UC ^[33,34]^, Graves’ disease ^[35,36]^
DUSP22	*DUSP22*	PTP	SLE ^[37]^, RA ^[38,39]^, IBD ^[40,41]^, psoriasis ^[42]^ Ankylosing spondylitis ^[43]^
CD45	*PTPRC*	PTP	SLE ^[44]^
DUSP2	*DUSP2*	PTP	RA ^[45]^, UC ^[46]^

CD, Crohn’s disease; DUSP2, dual specificity phosphatase 2; DUSP22, dual specificity phosphatase 22; IBD, inflammatory bowel disease; MS, multiple sclerosis; PP2A, protein phosphatase 2A; PSP, protein serine/threonine phosphatases; PTP, protein tyrosine phosphatases; PTPN22, PTP non-receptor type 22; RA, rheumatoid arthritis; SHP1, Src homology-2 domain-containing PTP1; SHP2, Src homology-2 domain-containing PTP2; SLE, systemic lupus erythematosus; TC-PTP, T-cell PTP; T1D, type 1 diabetes; UC, ulcerative colitis.

## 2. Phosphatase signaling in autoimmune diseases

### 2.1 PP2A

#### 2.1.1 PP2A composition

**Figure 1.**
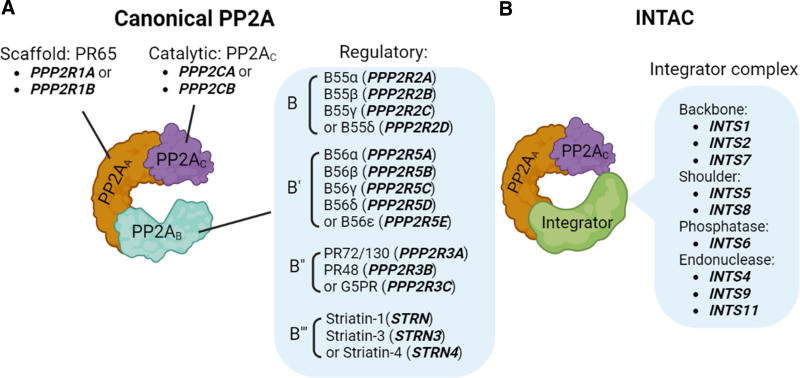
**The composition of PP2A holoenzyme.** (A) Canonical PP2A complexes are composed of The PP2A_A_ (scaffold subunit), PP2A_C_ (catalytic subunit), and PP2A_B_ (regulatory subunit). (B) The Integrator-PP2A complex (INTAC) consists of the PP2A core enzyme (PP2A_A_ and PP2A_C_) and nine subunits from the integrator complex. PP2A, protein phosphatase 2A.

PP2A is a ubiquitously expressed and highly conserved PSP that is important in multiple cellular processes including cell division, cytoskeletal dynamics, and various signaling pathways ^[47]^. The PP2A core enzyme consists of the scaffold subunit A (PP2A_A_, encoded by *PPP2R1A* or *PPP2R1B*) and the catalytic subunit C (PP2A_C_, encoded by *PPP2CA* or *PPP2CB*). To form a canonical PP2A functional holoenzyme, the core enzyme interacts with one of the many regulatory subunits (PP2A_B_), which define substrate and tissue specificity (Figure 1A) ^[48]^. The regulatory subunits are classified into four major families, identified as B (eg, PPP2R2A, PPP2R2B, PPP2R2C, or PPP2R2D), B’ (PPP2R5A, PPP2R5B, PPP2R5C, PPP2R5D, or PPP2R5E), B” (PPP2R3A, PPP2R3B, or PPP2R3C) and B’’’ (STRN, STRN3, or STRN4) (Figure 1A) ^[48]^. Recently, a non-canonical PP2A holoenzyme termed Integrator-PP2A complex (INTAC) was reported ^[49]^. Cryogenic electron microscopy structure studies revealed that the INTAC consists of the core enzyme of PP2A and nine subunits of the integrator complex, which replaces the PP2A_B_ subunit of the canonical PP2A holoenzyme (Figure 1B) ^[49]^. The integrator complex comprises distinct modules: the backbone (INTS1, INTS2, and INTS7), shoulder (INTS5 and INTS8), phosphatase (INTS6), and endonuclease (INTS4, INTS9, and INTS11) (Figure 1B) ^[49]^. The INTAC targets RNA polymerase II and the components of the basal transcription machinery to regulate gene transcription ^[50]^.

### 2.2 PP2A in autoimmune disorders

#### 2.2.1 Clinical evidence

**Figure 2.**
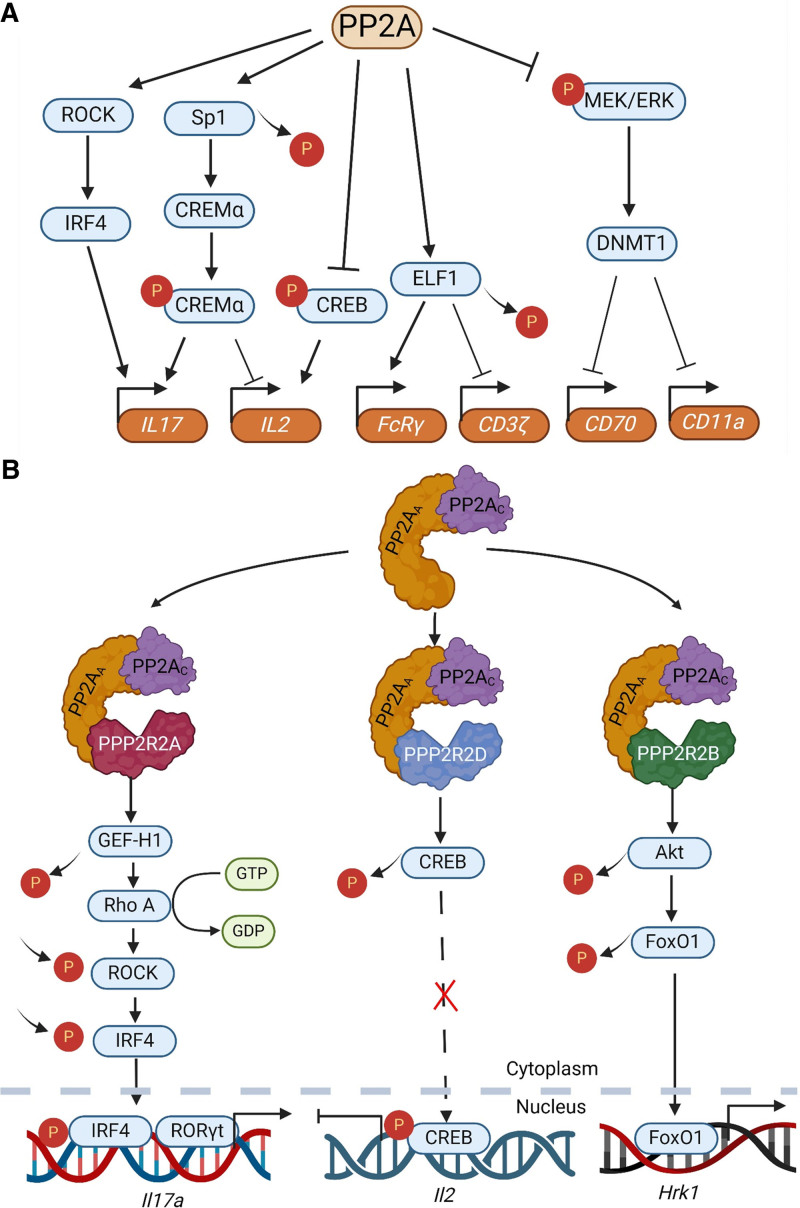
**The role of PP2A in T-cells.** (A) PP2A regulates SLE T-cell signaling. PP2A enhances ROCK activity, leading to phosphorylation of IRF4 that promotes IL-17 transcription. PP2A dephosphorylates and activates SP1 to increase the expression of CREMα which enhances the expression of IL-17 and represses expression of IL-2. PP2A also dephosphorylates CREB and limits the transcription of IL-2 resulting in decreased production of IL-2. PP2A dephosphorylates and inhibits ELF1 which inhibits CD3*ζ* transcription while enhances the transcription of FcRγ. PP2A inhibits the phosphorylation of MEK/ERK to block the activity of the methyltransferase DNMT1, resulting in hypomethylation and increased expression of CD70 and CD11a. (B) PP2A regulatory subunits control distinct functions in T-cells. PPP2R2A enhances GEF-H1/RhoA/ROCK signaling to promote IL-17 expression. PP2A_C_ associates with PPP2R2D to dephosphorylate CREB, leading to epigenetic closure of the IL-2 locus and inhibition of IL-2 expression. PP2A_C_ associates with PPP2R2B and dephosphorylates AKT to block the phosphorylation of FOXO1 which promotes the transcription of the proapoptotic molecule Hrk. CREB, cAMP response element-binding protein; CREMα, cAMP-responsive element modulatorα; ELF1, E74-like factor 1; GEF-H1/RhoA/ROCK, guanine nucleotide exchange factor H1/Ras homolog family member A/ROCK; IL, interleukin; PP2A, protein phosphatase 2A; SLE, systemic lupus erythematosus;

During the past two decades, multiple roles of PP2A in systemic lupus erythematosus (SLE) have been uncovered (Figure 2A). In 2005, increased levels of PP2A_C_ mRNA, protein, and catalytic activity were identified in human SLE T cells, but not in cells from other rheumatic diseases, leading to the dephosphorylation of cAMP response element-binding protein (CREB) and limiting interleukin (IL)-2 transcription and production ^[6]^. Subsequently, an intronic single-nucleotide polymorphism (SNP) in the first intron of *PPP2CA* was found to be linked to SLE, and patients with SLE who carried the SNP had higher PP2A_C_ expression in their T cells ^[7]^. Thus, *PPP2CA* (PP2A_C_) is characterized as a lupus susceptibility gene. Epigenetic modulation through methylation of CpG motifs in the *PPP2CA* promoter accounts for its increased expression ^[8]^. Further studies showed that PP2A dephosphorylates and inhibits Elf1, the main transcriptional enhancer of *CD3*ζ ^[9]^, whereas it enhances the transcription of *FcR*γ, a gene for which E74-like factor 1 (ELF1) represents a repressor ^[10]^. Thus, increased PP2A levels in SLE T cells can, at least partially, explain the decreased expression of CD3*ζ* and the enhanced expression of FcRγ in these cells ^[11]^. Moreover, PP2A dephosphorylates and activates SP1, a factor involved in the transcription of cAMP-responsive element modulator-α in SLE T cells ^[12]^. PP2A also regulates the activity of DNA methyltransferase (DNMT)1 in healthy control and SLE T cells ^[13]^.

**Figure 3.**
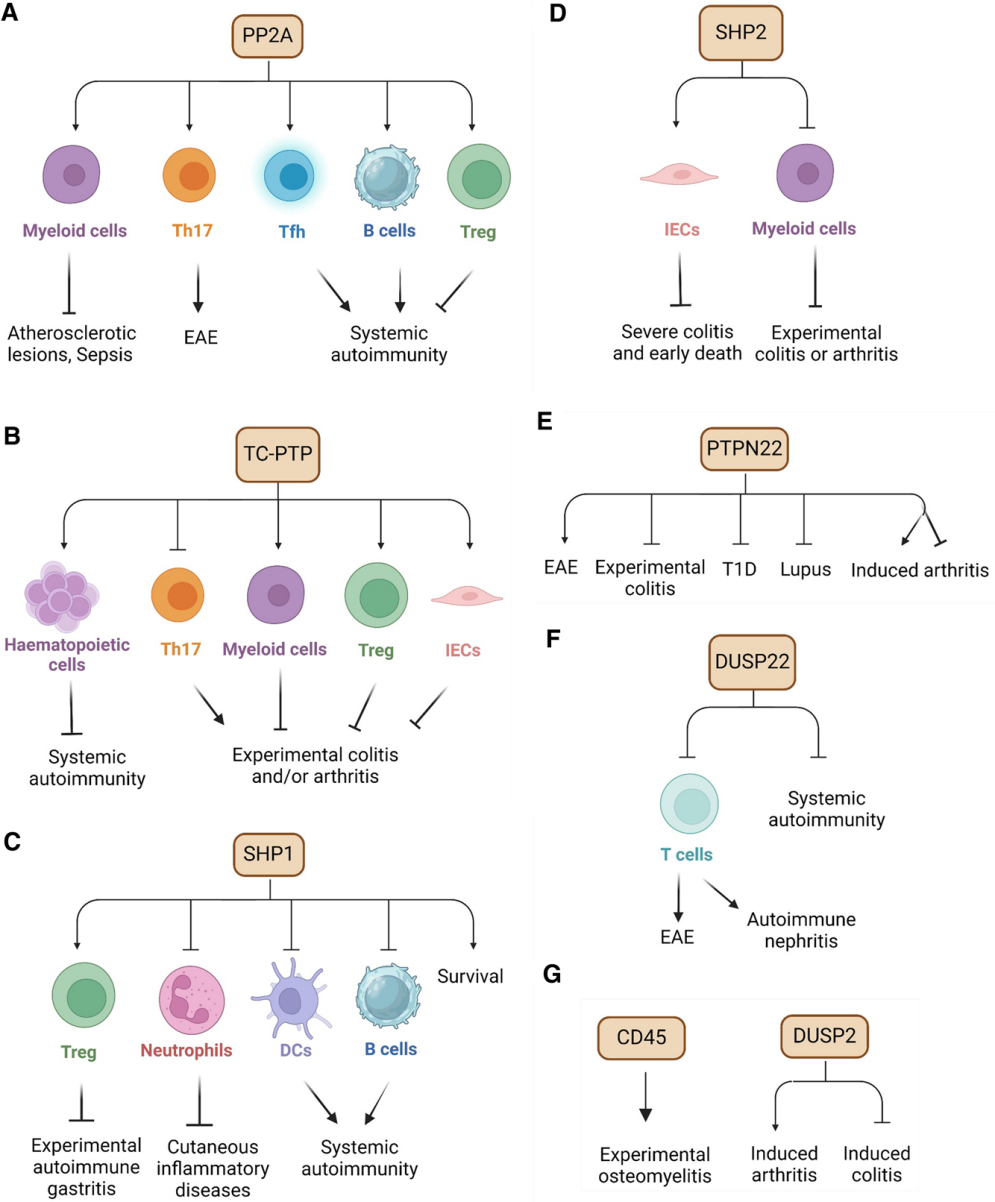
**The roles of phosphatases in autoimmune disorders.** The contribution of phosphatase PP2A (A), TC-PTP (B), SHP1 (C), SHP2 (D), PTPN22 (E), DUSP22 (F), CD45 and DUSP2 (G) in the pathogenesis and development of autoimmune disorders. Their roles have been validated in autoimmune models as indicated. DCs, dendritic cells; DUSP2, dual specificity phosphatase 2; DUSP22, dual specificity phosphatase 22; EAE, experimental autoimmune encephalomyelitis; IECs, intestinal epithelial cells; PP2A, protein phosphatase 2A; PTPN22, protein tyrosine phosphatases non-receptor type 22; SHP1, Src homology-2 domain-containing PTP1; SHP2, Src homology-2 domain-containing PTP2; T1D, type I diabetes; TC-PTP, T-cell PTP.

A number of genetically modified mouse models have been used to characterize the role of PP2A in autoimmunity (Figures 2B and 3A).

#### 2.2.2 PP2A is essential for thymocyte development

Loss of PP2A_C_ increases the phosphorylation of apoptosis-related proteins in double-positive (DP) cells, promoting thymocyte apoptosis ^[51]^. Thymic-specific depletion of PP2A_C_ in mice results in aberrant thymocyte development with noticeably decreased numbers of DP cells ^[51]^.

#### 2.2.3 PP2A is important for myeloid-cell function

PP2A activity is decreased in human atherosclerotic arteries ^[52]^. Myeloid-cell-specific deletion of PP2A exacerbates atherosclerotic lesions in mice fed with a high-fat, high-cholesterol diet ^[52]^, and amplifies MyD88- and TIR-domain-containing adapter-inducing interferon-β (TRIF)-dependent inflammation following endotoxin- and bacteria-induced sepsis in mice ^[53]^.

#### 2.2.4 PP2A promotes IL-17 production and T_H_17 cell differentiation

PP2A_C_ overexpression in T cells of mice leads to autoimmunity and lupus-like pathology in an IL-17-dependent manner, when challenged with an anti-glomerular basement membrane antibody ^[54]^. Mechanistic studies showed that PP2A enables the expression of IL-17 through chromatin remodeling ^[55–63]^. In contrast, PP2A_C_ deficiency impairs the differentiation of naïve CD4 T cells into T_H_17 cells by changing the phosphorylation status of receptor-regulated Suppressor of Mothers against Decapentaplegics ^[64]^. Mice lacking PP2A_C_ expression in T cells display decreased T_H_17 cell numbers and less severe disease in an experimental autoimmune encephalomyelitis (EAE) model ^[64]^.

#### 2.2.5 PP2A is critical for follicular T helper (T_FH_) cell development and function

Ablation of PP2A_C_ inhibits the differentiation and function of T_FH_ cells by downregulating BCL6 expression in vitro ^[65]^. Mice with T-cell-specific depletion of PP2A_C_ show reduced T_FH_ cell differentiation, germinal center (GC) response, and autoantibody production after immunization with sheep red blood cells or chromatin ^[65]^. PP2A_C_ ablation in T cells also mitigates systemic autoimmunity in lupus-prone MRL/*lpr* mice ^[65]^.

#### 2.2.6 PP2A is vital for optimal human and murine B cell function

PP2A activity is increased in activated B-cells and B-cells from lupus-prone mice and in patients with SLE ^[66]^. Ablation of PP2A activity in B-cells through depletion of PPP2R1A impedes B-cell differentiation and activation in response to stimulation in vitro. Mice with B-cell-specific depletion of PPP2R1A display less autoimmunity with reduced spontaneous GC formation, lower titers of anti-ANA IgG in the serum, and decreased IgG deposition in the kidneys in a pristane-induced lupus model ^[66]^.

#### 2.2.7 PP2A is requisite for the function of regulatory T (Treg) cells

Ablation of functional PP2A through depletion of PPP2R1A reduces regulatory T (Treg) cell suppressive function by increasing the activity of mammalian target of rapamycin complex 1 ^[67]^. Treg-cell-specific ablation of PPP2R1A in mice causes a severe, multi-organ, lymphoproliferative autoimmune disorder ^[67]^. Further studies show that PP2A maintains Treg cell development and function by blocking the sheddase activity of ADAM10, which dampens IL-2 receptor signaling by cleaving IL-2Rβ ^[68]^. In human Treg cells, PP2A and CD25 (IL-2Rα) cooperatively enhance the responsiveness of Treg cells to IL-2 ^[69]^.

#### 2.2.8 PP2A regulatory subunits control specific immune cell functions

PPP2R2B appears to trigger apoptosis in T cells in the absence of IL-2 by modulating AKT and the proapoptotic molecule Hrk, probably contributing to the termination of a no-longer-needed immune response (Figure 2B) ^[70,71]^.

PPP2R2D, which is increased in SLE T cells, has been shown to suppress IL-2 production and Treg cell function by dephosphorylating CREB and promoting epigenetic closure of the IL-2 locus, as demonstrated in studies using genetically engineered mice and human samples ^[72]^. T-cell-specific depletion of PPP2R2D alleviates systemic autoimmunity after exposure to a TLR7 stimulator ^[72]^, and advances tumor growth ^[73]^ in mice.

PPP2R2A, which is also increased in T cells from patients with SLE, enhances T_H_1 and T_H_17 cell differentiation through activation of the guanine nucleotide exchange factor H1/Ras homolog family member A/ROCK (GEF-H1/RhoA/ROCK) signaling pathway ^[74]^. T-cell-specific deletion of PPP2R2A reduced T_H_17 cell numbers and autoimmune syndrome development in an EAE model ^[74]^ and lupus-prone mice ^[75]^.

## 3. TC-PTP

### 3.1 TC-PTP composition

TC-PTP, encoded by the *PTPN2* gene, is a ubiquitously expressed non-receptor PTP. There are two primary isoforms of TC-PTP due to alternative splicing. The 45 kDa isoform (TC45) contains a bipartite nuclear localization signal that directs it to the nucleus. In response to stimuli, TC45 shuttles to the cytoplasm, where it interacts with substrates either in the cytoplasm or at the plasma membrane. The 48 kDa isoform (TC48) contains a hydrophobic *C*-terminal sequence that targets it to the endoplasmic reticulum (ER). Unlike TC45, TC48 remains more restricted to the ER, which limits its access to certain substrates ^[76]^. The functional differences between these isoforms allow TC-PTP to have distinct roles in various cellular compartments, influencing signaling pathways and the regulation of immune responses.

### 3.2 TC-PTP in autoimmune disorders

#### 3.2.1 Clinical evidence

The *PTPN2* gene, which encodes TC-PTP, appears to be crucial in regulating immune responses, and certain polymorphisms in this gene can elevate the risk for autoimmunity ^[77]^. The *rs2542151* SNP was first reported to be linked with Crohn’s disease (CD) ^[14]^ and further studies showed that it is also associated with ulcerative colitis (UC) ^[16]^. The *rs1893217* SNP, while originally identified as a genetic risk factor for type 1 diabetes (T1D) and celiac disease ^[17,18]^, has also been implicated in the pathogenesis of CD and UC ^[14]^. In addition, it downregulates TC-PTP protein levels leading to impaired IL-2/IL-2R signaling in CD4 T cells ^[15]^. Recently, six distinct heterozygous *PTPN2* mutations (F403Lfs*25, W98*, C216S, E24Mfs*20, W289*, and Y126N), which result in loss of PTPN2 regulatory function, were reported to associate with patients with Evans syndrome or pediatric-onset systemic lupus ^[78]^.

#### 3.2.2 TC-PTP is crucial for maintaining immune homeostasis

Mice with global homozygous genetic deletion of TC-PTP exhibit severe inflammatory disease and die within 3–4 weeks after birth ^[79]^. Global heterozygous TC-PTP deletion (partial loss of TC-PTP expression) exacerbates experimental colitis ^[80]^ and inflammatory arthritis in the SKG mouse model of autoimmune arthritis ^[81]^. When TC-PTP is inducibly deleted specifically in the hematopoietic system of adult mice, they develop systemic inflammation and autoimmunity (Figure 3B) ^[82]^.

#### 3.2.3 TC-PTP restrains T helper cell induction and function

T-cell-specific TC-PTP deficiency in non-obese diabetic mouse (NOD) mice accelerates the onset and increases the incidence of T1D, colitis, and Sjögren syndrome, accompanied by an increase in T_H_1, T_FH_, and B cells in the pancreatic islets ^[83]^. In addition, mice with T-cell-specific loss of TC-PTP exhibit elevated T_H_1 and T_H_17 cell numbers and autoantibody levels, leading to intestinal inflammation, dysbiosis, and multi-organ inflammatory infiltrates in several colitis models ^[84]^. T-cell-specific heterozygous deletion of TC-PTP promotes arthritis in SKG mice in an IL-6- and IL-17-dependent manner ^[51]^.

#### 3.2.4 TC-PTP is essential for inhibiting Treg cell plasticity

Treg-cell-specific heterozygous deletion of TC-PTP exacerbates arthritis in SKG mice ^[81]^. A mechanistic study revealed that loss of TC-PTP expression in Treg cells enhances IL-6-induced signal transducer and activator of transcription (STAT)3 phosphorylation, driving the conversion of Treg cells into pathogenic IL-17-producing “exTreg” cells ^[81]^. In addition, a study shows that TC-PTP interacts with tumor necrosis factor receptor associated factor (TRAF)3 to dampen the development of Treg cells by inhibiting IL-2/STAT5 signaling ^[85]^. Also, inducible Treg-cell-specific heterozygous deletion of TC-PTP worsens colitis-induced SKG arthritis and leads to specific accumulation of GPR15+ exTreg cells in the joints ^[86]^.

#### 3.2.5 TC-PTP is required for interactions between macrophages and intestinal epithelial cells

Myeloid-cell-specific deletion of TC-PTP in mice enhances IL-1β-dependent intestinal inflammation ^[87]^ and increases intestinal epithelial cell (IEC) monolayer permeability and the development of inflammatory macrophages in an IL-6-dependent manner ^[88]^. IEC-specific ablation of TC-PTP aggravates experimental colitis in mice ^[89]^.

## 4. SHP1

### 4.1 SHP1 composition

SHP1, encoded by *PTPN6*, is a non-receptor PTP that has two distinct isoforms. One isoform is primarily expressed in hematopoietic cells and is localized in the cytosol, whereas the other is expressed in epithelial cells and localizes to the nucleus ^[90]^. This differential localization suggests that the two isoforms of SHP1 may target different substrates and have distinct cellular functions ^[90]^. SHP1 consists of a PTP catalytic domain, a *C*-terminal region with Tyr phosphorylation sites, and two tandem Src homology-2 (SH2) domains, N-SH2 and C-SH2 ^[90]^. The N-SH2 domain blocks substrate access to the catalytic domain and keeps the enzyme in an inactive conformation. When a phospho-Tyr-containing peptide is recruited by the N-SH2 or C-SH2 domain, it activates SHP1 ^[91,92]^.

### 4.2 SHP1 in autoimmune disorders

#### 4.2.1 Clinical evidence

SHP1 plays an important regulatory role in immune tolerance and has been linked to several autoimmune disorders. SHP1 deficiency is found in peripheral blood mononuclear cells (PBMCs) from patients with multiple sclerosis (MS) ^[19]^. In addition, peripheral monocyte-derived macrophages from patients with MS express low SHP1, and depletion of SHP1 in macrophages promotes STAT/nuclear factor kappa-light-chain-enhancer of activated B cells (NF-kB) activation and increases inflammatory gene expression ^[20]^. In T cells from patients with rheumatoid arthritis (RA), enhanced ERK activity delays the recruitment of SHP1 to the T cell receptor (TCR)-antigen-presenting cell synapse ^[21]^. SHP1 with an Ala455Thr mutation, which leads to a reduction of its phosphatase activity, promotes early-onset emphysema, and reduces diffusion capacity in a large French-Canadian family ^[22]^.

#### 4.2.2 SHP1 is vital for maintaining immune homeostasis

Mice with homozygous *motheaten (me/me*) mutation at the *PTPN6* gene locus that leads to SHP1 deficiency develop severe autoimmune and immunodeficiency syndromes, resulting in death two or three weeks after birth ^[90,93]^. Homozygous *motheaten viable (me*^*v*^*/me*^*v*^) mutation which causes partial depletion of SHP1, also induces similar but less severe autoimmunity and increases the life span to 8–12 weeks in mice ^[90,94,95]^. Heterozygous *me*^*v*^*(+/−*) mutation induces autoreactive T cells and more severe disease in an EAE mouse model ^[96]^. In addition, mice with a hypomorphic allele of *PTPN6* (m1Btlr, designated spin), which causes a homozygous Tyr208Asn amino acid mutation in SHP1, develop neutrophilic dermatosis-like chronic inflammatory and autoimmune disease, which depends on IL-1/MyD88 ^[97]^, and ASK1/2 signaling ^[98]^. In contrast, transgenic mice with homozygous SHP1 overexpression are resistant to arthritis after immunization (Figure 3C) ^[99]^.

#### 4.2.3 SHP1 enhances Treg cell proliferation and mobility

Treg cells with loss of SHP1 expression show reduced proliferation in response to IL-2 stimulation ^[100]^. Treg-cell-specific deletion of SHP1 impairs the control of inflammation in mouse models of allergic airway inflammation, and autoimmune gastritis in DEREG (DEpletion of REGulatory T cells) mice, probably due to defective proliferation or migration of SHP1-deficient Treg cells to peripheral inflammation sites ^[100]^.

#### 4.2.4 SHP1 is important for B-cell response to stimulation

Young mice with loss of SHP1 specifically in B cells show a marked reduction in GC B cell frequencies ^[101]^, and impaired antibody responses ^[102]^ after immunization. However, older (5–11 month) mice with B cell-specific SHP1 deficiency develop systemic autoimmunity, including lymphadenopathy in the spleen and lymph nodes, elevated serum autoantibodies, and immune complex-mediated glomerulonephritis ^[102]^.

#### 4.2.5 SHP1 is a key regulator of dendritic cell (DC) function

Mice lacking SHP1 specifically in dendritic cells (DCs) exhibit increased numbers of DCs, B, and T_H_1 cells in the spleen, and elevated levels of IgM and IgG2a in the serum ^[103]^. In addition, aged mice (36–40 weeks) with DC-specific SHP1 deletion develop systemic autoimmunity, including increased autoantibodies in the serum, glomerulonephritis, and interstitial pneumonitis due to exaggerated MyD88-dependent signaling ^[103,104]^.

#### 4.2.6 SHP1 is a key regulator of neutrophil function

Although neutrophil-specific depletion of SHP1 does not lead to autoimmunity, the mice manifest a spontaneous cutaneous inflammatory disease by enhanced neutrophil integrin signaling through Src-family and Syk kinases ^[104]^, or by promoting the secretion of TNF and IL-1α/β from neutrophils through caspase-8- and Ripk3/Mlkl-dependent pathways ^[105]^. In addition, neutrophil-specific deletion of SHP1 leads to Syk kinase-dependent hyperinflammation and lethal pulmonary hemorrhage in lipopolysaccharide- or pathogens-induced acute lung injury models ^[106]^.

## 5. SHP2

### 5.1 SHP2 composition

SHP2, encoded by the *PTPN11* gene, is a non-receptor PTP, which is ubiquitously expressed in various cell types and tissues. Like its homolog SHP1, SHP2 is composed of a PTP catalytic domain, a *C*-terminal region with Tyr phosphorylation sites, and two tandem SH2 domains (N-SH2 and C-SH2) ^[90,107]^. SHP2 activity, similar to SHP1, is regulated by an auto-inhibitory mechanism. In its inactive form, the N-SH2 domain blocks the catalytic site of the PTP domain, preventing substrate access. Upon interaction with phosphorylated tyrosine residues on target proteins, the N-SH2 domain undergoes conformational changes, relieving the inhibitory effect and activating the phosphatase activity of SHP2 ^[90,108,109]^.

### 5.2 SHP2 in autoimmune disorders

#### 5.2.1 Clinical evidence

SHP2 is involved in regulating immune cell function and has been identified as a key regulator in autoimmune diseases. The *PTPN11* gene is implicated in the genetic susceptibility to RA because it is located within a linkage disequilibrium block that is associated with RA ^[23]^. Polymorphisms in the *PTPN11* gene have also been linked to increased susceptibility to UC and CD ^[14,24]^. Rare individuals with missense mutations causing SHP2 hyperactivity develop the Noonan syndrome and invariably present with SLE-like clinical manifestations ^[25,26]^. Lymphocytes from SLE patients have increased levels of SHP2 ^[26]^. Increased SHP2 expression was also found in colonic macrophages and blood monocytes from patients with inflammatory bowel disease (IBD) ^[27]^.

Only a small number of studies using SHP2 genetically engineered mice in autoimmune disorders have been reported (Figure 3D). Myeloid-cell-specific deletion of SHP2 protects mice from acute colitis through IL-10-mediated anti-inflammatory function and signaling in macrophages ^[27]^ and reduces inflammatory arthritis in mice ^[110]^. In contrast, mice with an IEC-specific PTPN11 deletion rapidly develop severe colitis and die as early as 3 to 4 weeks after birth ^[111]^.

## 6. PTPN22

### 6.1 PTPN22 composition

PTPN22, also known as lymphoid tyrosine phosphatase, is encoded by the *PTPN22* gene and is a non-receptor PTP expressed in hematopoietic cells. PTPN22 consists of an *N*-terminal PTP catalytic domain, an interdomain region, and a *C*-terminal segment with four proline-rich sequence motifs, labeled P1 to P4, in which the P1 motif interacts with the SH3 domain of *C*-terminal Src family kinase ^[112]^.

### 6.2 PTPN22 in autoimmune disorders

#### 6.2.1 Clinical evidence

Two SNPs at the *PTPN22* gene locus have been strongly associated with several autoimmune diseases, and their contribution to disease risk is context-dependent ^[77,113]^. The *rs2476601* SNP results in the substitution of an arginine with a tryptophan residue at position 620 of the PTPN22 protein (R620W variant), which increases the phosphatase activity of PTPN22 ^[114]^. This variant elevates the risk of autoimmune disorders, including T1D ^[28]^, SLE ^[29]^, RA ^[29,31]^, UC ^[33]^, and autoimmune thyroid diseases (Graves’ disease) ^[35,36]^. Functional studies have shown that the R620W variant reduces T or B cell responses to TCR or BCR stimulation, and individuals with this variant have fewer memory T and B cells ^[115,116]^, but higher numbers of mildly self-reactive T cells ^[117]^ and autoreactive B cells ^[118,119]^. In contrast, the *rs33996649* SNP in which arginine is substituted with a glutamine residue at position 263 within the catalytic domain (R263Q variant) decreases the phosphatase activity ^[30]^ of PTPN22 and reduces the risk of developing SLE ^[30]^, RA ^[32]^, and UC ^[34]^. Collectively, these findings suggest that PTPN22 promotes the development of autoimmune diseases.

#### 6.2.2 PTPN22 regulates context-dependent immune homeostasis

Mice with homozygous deletion of PTPN22 remain healthy without developing spontaneous autoimmunity but exhibit expanded effector/memory T cell populations, spontaneous GC development, elevated antibodies in the serum ^[120,121]^, and increased Treg cell numbers with enhanced suppressive and adhesive functions ^[122]^. However, when encountering specific autoimmune challenges, the PTPN22-deficient mice have distinct outcomes depending on the disease context. In an EAE model, loss of PTPN22 reduces disease severity, probably due to the expanded Treg cell population ^[123]^. In a dextran sulfate sodium-induced colitis model, PTPN22 deficiency exacerbates colitis ^[123–125]^. In a spontaneous T1D NOD mouse model, the introduction of the murine orthologous Ptpn22(R619W) mutation elevates insulin autoantibodies and accelerates the onset of T1D ^[126]^. In mice of nonautoimmune-prone background, PTPN22 ablation increases interferon (IFN)-α-induced lupus-like disease ^[127]^, while in lupus-prone mice, it exacerbates the severity of systemic autoimmunity ^[128,129]^. In arthritis models, PTPN22 deficiency reduces disease severity in the arthritogenic serum-induced arthritis ^[130]^ and autoimmune arthritis SKG mouse models ^[131]^, while it accelerates the development of arthritis in the KBxN mouse ^[131]^. These findings from PTPN22-deficient or mutant mice highlight the complex and context-dependent role of PTPN22 in immune regulation. Cell-type-specific deletion of PTPN22 in mice is warranted to further elucidate its role in regulating autoimmune disorders (Figure 3E).

## 7. DUSP22

### 7.1 DUSP22 composition

DUSP22, also known as c-Jun *N*-terminal kinase (JNK) pathway-associated phosphatase and encoded by the *DUSP22* gene, is a small PTP comprising 184 amino acids and is ubiquitously expressed in various cell types and tissues ^[132,133]^. DUSP22 mediates non-membrane-spanning PTP activity, regulates the activity of various protein kinases, and specifically activates the c-Jun *N*-terminal kinase pathway ^[134,135]^.

### 7.2 DUSP22 in autoimmune disorders

#### 7.2.1 Clinical evidence

Reduced protein levels of DUSP22 were found in T cells from patients with SLE and inversely correlated with SLE disease activity ^[37]^. Serum DUSP22 levels are decreased in patients with psoriasis and negatively correlate with the psoriasis area severity index ^[42]^. *DUSP22* mRNA levels are low in T cells from patients with ankylosing spondylitis ^[43]^. Synovium and serum DUSP22 levels decrease in patients with RA and negatively correlate with systemic inflammation, disease activity, and joint dysfunction ^[38,39]^. Additionally, DUSP22 expression is decreased in the serum or inflamed mucosa of patients with active IBD and negatively correlates with disease activity ^[40,41]^.

#### 7.2.2 DUSP22 is necessary for maintaining immune homeostasis

Several mouse studies on DUSP22 in autoimmune disorders have been reported. Transgenic mice with T-cell-specific expression of a dominant-negative DUSP22 mutant develop spontaneous autoimmune nephritis ^[37]^. While young DUSP22-deficient mice appear normal, aged (1-year-old) DUSP22 knockout mice develop spontaneous inflammation and autoimmunity ^[43,136]^. DUSP22-deficient T cells exhibit enhanced cell proliferation and cytokine production, and DUSP22 global knockout mice exhibit accelerated disease symptoms in EAE models (Figure 3F) ^[136]^.

## 8. Other phosphatase in autoimmune disorders

Aside from the phosphatases mentioned above, two other tyrosine phosphatases have been implicated in the development of autoimmunity, although in limited studies (Figure 3G).

### 8.1 CD45

CD45, encoded by the *PTPRC* gene, is a transmembrane protein that functions as a receptor PTP and is exclusively expressed on hematopoietic cells. The phosphatase activity of CD45 was found to decrease in peripheral blood lymphocytes from patients with SLE ^[44]^. In an autoinflammatory osteomyelitis mouse model, CD45 deficiency inhibits the onset and severity of bone inflammation by inactivating Src family kinases, which enhance IL-1β-mediated inflammatory signaling ^[137]^.

### 8.2 DUSP2

DUSP2, also known as phosphatase of activated cells 1 (PAC1) and encoded by the *DUSP2* gene, localizes to the nucleus and dephosphorylates both phospho-Ser/Thr and phospho-Tyr residues ^[138,139]^. The DUSP2 protein is highly expressed in human RA synovium and tonsil and global knockout of DUSP2 in mice alleviates disease pathogenesis in the KBxN model of inflammatory arthritis ^[45]^. However, *DUSP2* mRNA levels are reduced in PBMCs from patients with UC. Loss of DUSP2 expression promotes T_H_17 cell differentiation in vitro and exacerbates experimental colitis in mice, which is associated with increased T_H_17 cell numbers and secretion of proinflammatory cytokines ^[46]^.

## 9. Targeting phosphatases in autoimmune diseases

The outlined evidence suggests that phosphatases play a key role in regulating immune responses, and their dysregulation can contribute to autoimmune diseases by altering immune tolerance and promoting inappropriate immune responses. Thus, targeting phosphatases has emerged as a promising therapeutic strategy for autoimmune disorders. Several inhibitors and activators of phosphatases (described below) have been evaluated in preclinical models or clinical trials, demonstrating potential efficacy in modulating immune responses and autoimmune conditions (Table 2).

**Table 2:** Modulators of protein phosphatases in treating autoimmune diseases.

Modulator	Target	Dose/frequency/route	Treatment subject
Inhibitor			
Cantharidin	PP2A	0.5 mg/kg/2 days i.p.	MRL/*lpr* mice ^[65]^
Cyclosporine	Calcineurin	2.5–10 mg/kg daily	Patients with RA ^[140]^ or psoriasis ^[141]^
Voclosporin	Calcineurin	23.7 mg twice daily	Patients with lupus nephritis ^[142,143]^
11a-1	SHP2	7.5 mg/kg daily i.p	Induced-arthritis model ^[110]^, and MRL/*lpr* mice ^[26]^
SHP099	SHP2	10 mg/kg daily orally	Imiquimod- or IL-23-induced psoriasis in mice ^[144]^
TK-453	SHP2	1–10 mg/kg daily	Imiquimod-induced psoriasis in mice ^[145]^
Activator			
FTY720	PP2A	1.25 or 5.0 mg orally, daily 2 mg/kg 3 times/week, daily	Patients with MS ^[146,147]^ MRL/*lpr* mice ^[148]^
Spermidine	TC-PTP	0.1 M solution in drinking water	Experimental colitis model ^[149]^
SC-43	SHP1	50 mg/kg orally, daily 10 mg/kg orally, daily	Bleomycin-induced mouse lung fibrosis model ^[150]^ LPS-induced acute lung injury model ^[106]^

LPS, lipopolysaccharide; MS, multiple sclerosis; PP2A, protein phosphatase 2A; RA, rheumatoid arthritis; SHP1, Src homology-2 domain-containing PTP1; SHP2, Src homology-2 domain-containing PTP2; SLE, systemic lupus erythematosus; TC-PTP, T-cell PTP;

## 10. Inhibitors

### 10.1 Cantharidin

Cantharidin, a natural compound found in the beetles of the *Meloidae* family, is a selective small molecule inhibitor of PP2A that disrupts its activity ^[151,152]^. In lupus-prone MRL/*lpr* mice, intraperitoneal (i.p.) injection of cantharidin (0.5 mg/kg/2 days) for 8 weeks improves survival, lowers serum levels of anti-dsDNA antibodies, and reduces proteinuria and immune complex deposition in the glomeruli ^[65]^.

### 10.2 Cyclosporine/voclosporin

Calcineurin is a calmodulin-dependent PSP ^[153]^ and is highly expressed in synoviocytes of patients with RA ^[154]^.

Cyclosporine binds to cyclophilin-1 within cells, forming a cyclosporine-cyclophilin complex that inhibits the activation of the phosphatase calcineurin ^[155]^. FDA-approved, oral administration of an ophthalmic formulation of cyclosporine (daily dose 2.5–10 mg/kg) improves clinical parameters in patients with severe and active RA ^[140]^ and in patients with severe and recalcitrant plaque psoriasis ^[141]^.

Voclosporin, a semisynthetic structural analog of cyclosporine, is a calcineurin inhibitor that blocks calcineurin activation ^[156]^. Voclosporin, sold under the brand name Lupkynis, is an immunosuppressant used for the treatment of lupus nephritis approved by the FDA in 2021 ^[142]^. In a Phase 3 clinical trial, oral administration of voclosporin (23.7 mg twice daily) resulted in a significantly higher complete renal response rate compared to the placebo group in patients with lupus nephritis ^[143]^.

### 10.3 SHP2 inhibitors

Several inhibitors targeting SHP2 have shown efficacy in alleviating autoimmunity in preclinical models.

#### 10.3.1 11a-1

11a-1 is a potent, selective, and cell-active SHP2 inhibitor that binds to the SHP2 active site ^[157]^. Daily treatment with 11a-1 (7.5 mg/kg i.p.) reduces the severity of inflammatory arthritis in an induced-arthritis model ^[110]^ and improves clinical and immunologic outcomes in MRL/*lpr* mice ^[26]^.

#### 10.3.2 SHP099

SHP099 is an allosteric inhibitor of SHP2. Daily SHP099 administration (10 mg/kg) ameliorates imiquimod- or IL-23-induced psoriasis progression in mice ^[144]^.

#### 10.3.3 TK-453

TK-453 (daily dose 1–10 mg/kg), another SHP2 allosteric inhibitor, alleviates imiquimod-induced psoriasis-like skin inflammation in mice by inhibiting the IL-23/T_H_17 axis ^[145]^.

## 11. Activators

### 11.1 FTY720

FTY720 (Fingolimod) is an activator of PP2A. It binds to the PP2A inhibitor protein SET, causing it to dissociate from the catalytic subunit PP2A_C,_ thereby activating PP2A ^[158]^. In clinical trials, daily oral administration of FTY720 (1.25 mg or 5.0 mg) reduces the number of lesions and clinical disease activity in patients with relapsing MS ^[146]^ by increasing the ratio of Treg to T_H_1/T_H_17 cells ^[147]^. FTY720, approved by the FDA in 2010 as an anti-inflammatory drug for MS, also attenuates behavioral deficits in MRL/*lpr* mice when administered orally (2 mg/kg) three times per week ^[148]^.

### 11.2 Spermidine

Spermidine is a TC-PTP agonist that activates TC-PTP (TC45 isoform) ^[159]^. Instead of binding to the catalytic domain of TC-PTP, spermidine competes with the α1 integrin cytoplasmic tail for TC45 binding, relieving autoinhibition mediated by the TC45 C terminus ^[159,160]^. Oral administration of spermidine (0.1 M solution) reduces weight loss, colonic inflammation, and damage in an experimental colitis mouse model, probably due to the enhanced TC-PTP-dependent IFN-γ-induced phosphorylation of STAT1/3 and p38 MAPK and expression levels of ICAM-1, MCP-1, and IL-6 in monocytes ^[149]^.

### 11.3 SC-43

SC-43 is an SHP1 agonist that enhances SHP1 activity by destruction of the association between the *N*-SH2 domain and PTP catalytic domain of SHP1, triggering a conformational change of SHP1 and relieving its autoinhibition. SC-43 treatment (50 mg/kg, orally) ameliorates pulmonary fibrosis in a bleomycin-induced pulmonary fibrosis murine model ^[150]^. Oral administration (10 mg/kg) of SC-43 reduces lung inflammation in a lipopolysaccharide-induced acute lung injury model ^[106]^.

## 12. Conclusions and future perspectives

Although significant progress has been made in our understanding of the biochemistry, biology, relevance to autoimmunity disorders, and exploitation of phosphatase-directed drug development, considerable challenges remain. Compelling evidence from genetically modified mouse models is strengthening the case that the expression of TC-PTP and SHP1 in immune cells is crucial to maintain immune homeostasis, and efforts to activate or overexpress them may have potential therapeutic value for diseases such as RA, T1D, or IBD. However, further validation studies are required at this stage to ensure the effectiveness and specificity of selective targeting agents, first in autoimmune models, and then in patients. Extensive studies have characterized the diverse roles of PP2A across various immune cell subsets in the context of autoimmunity. Yet, there are apparent contradictory data on the role of phosphatases in various immune cell subsets and experimental settings. For example, PP2A is requisite for Treg function, but in parallel, it promotes T_H_17 cell differentiation, an opposite control of cell function. This makes PP2A inhibitors unlikely to be used for the treatment of patients with autoimmune disorders in which both cell types contribute to their pathogenesis. Nonetheless, there is evidence that individual PP2A regulatory subunits control distinct immune cell functions, such as IL-17 and IL-2 production and CD8 cell survival. Given that canonical PP2A holoenzyme has more than 20 regulatory subunits and the role of non-canonical PP2A in autoimmunity is barely known, it is important to further characterize their roles in the expression of autoimmune pathology.

Although PTPN22 has been implicated in the pathogenesis of multiple autoimmune diseases, global PTPN22-deficient or -mutant mice present either protective or harmful roles in the disease progression in mice in response to different autoimmune challenges. Therefore, PTPN22 inhibition could have diverse effects depending on the specific autoimmune condition. To resolve these uncertainties, further cell-specific studies using genetically modified mice with cell-specific deletion or overexpression of PTPN22 are needed.

Preliminary studies have indicated that DUSP22 activation and SHP2 inhibition may offer potentially therapeutic benefits for RA and IBD. However, these initial findings need further validation. At present, the evidence for the role of CD45 and DUSP2 in the pathogenesis of autoimmune disorders is limited, and further studies using conditional knockout mice are warranted.

Despite advances in developing phosphatase modulators, a deeper molecular understanding of the target and cell-based assays to confirm intracellular engagement and biological effects is crucial. Given that some phosphatases, such as PP2A, TC-PTP, SHP2, and DUSP22, are ubiquitously expressed in various cell types and tissues, targeted delivery of phosphatase inhibitors/activators to specific cell types using nanoparticles should limit side effects. Such an approach will be possible if drug-loaded nanoparticles are tagged with antibodies recognizing the specific cell surface markers.

## Author contributions

WP designed, conceived, and wrote the manuscript. MGT provided critical expertise and edited the manuscript. WL edited the manuscript. GCT conceived and edited the manuscript. All authors have read and approved the manuscript.

## Conflicts of interests

The authors declare that they have no conflicts of interest.

## Funding

This work was supported by NIH grant R01 AI136924.
